# Temporal analysis of cardiovascular control and function following incomplete T3 and T10 spinal cord injury in rodents

**DOI:** 10.14814/phy2.13634

**Published:** 2018-03-29

**Authors:** Kathryn A. Harman, Gregory States, Abigail Wade, Chad Stepp, Grace Wainwright, Kathryn DeVeau, Nicholas King, Alice Shum‐Siu, David S. K. Magnuson

**Affiliations:** ^1^ Anatomical Sciences and Neurobiology University of Louisville Louisville Kentucky; ^2^ Department of Neurological Surgery University of Louisville Louisville Kentucky; ^3^ Biomedical Engineering University of Louisville Louisville Kentucky

**Keywords:** Cardiovascular, exercise, spinal cord injury

## Abstract

Spinal cord injury (SCI) is a devastating condition that results in whole‐body dysfunction, notably cardiovascular (CV) disruption and disease. Injury‐induced destruction of autonomic pathways in conjunction with a progressive decline in physical fitness contribute to the poor CV status of SCI individuals. Despite the wide use of exercise training as a therapeutic option to reduce CV dysfunction, little is known about the acute hemodynamic responses to the exercise itself. We investigated CV responses to an exercise challenge (swimming) following both high and low thoracic contusion to determine if the CV system is able to respond appropriately to the challenge of swimming. Blood pressure (BP) telemetry and echocardiography were used to track the progression of dysfunction in rodents with T3 and T10 SCI (*n* = 8 each) for 10 weeks postcontusion. At 1 week postinjury, all animals displayed a drastic decline in heart rate (HR) during the exercise challenge, likely a consequence of neurogenic shock. Furthermore, over time, all groups developed a progressive inability to maintain BP within a narrow range during the exercise challenge despite displaying normal hemodynamic parameters at rest. Echocardiography of T10 animals revealed no persistent signs of cardiac dysfunction; T3 animals exhibited a transient decline in systolic function that returned to preinjury levels by 10 weeks postinjury. Novel evidence provided here illustrates that incomplete injuries produce hemodynamic instability that only becomes apparent during an exercise challenge. Further, this dysfunction lasts into the chronic phase of disease progression despite significant recovery of hindlimb locomotion and cardiac function.

## Introduction

Spinal cord injury (SCI) is a devastating life event that results in extreme somatic, autonomic, and sensory dysfunction. To date, the majority of clinical and preclinical SCI research has concentrated on the below‐lesion paralysis and recovery of locomotor function. However, secondary complications, most notably those of the autonomic nervous system, remain the leading causes of mortality and morbidity in the chronic SCI community. Specifically, cardiovascular disease (CVD) occurs earlier and is more robust in SCI individuals than in the able‐bodied counterparts, differences which cannot be solely explained by traditional risk factors (lipid profiles, age, obesity, smoking status, etc.) (Whiteneck et al. 1992; Cragg et al. 2012). Abnormal control of arterial blood pressure (BP) and heart rate (HR) can frequently lead to episodes of autonomic dysreflexia (AD) and orthostatic hypotension (OH), making activities of daily living difficult. In addition to maladaptive nervous system reorganization and plasticity following injury (Krenz and Weaver 1998; Hou et al. 2008, 2009), structural changes in the heart and vasculature contribute to aberrant cardiovascular (CV) regulation and unstable hemodynamics (Laird et al. 2008; Lujan et al. 2012; Thijssen et al. 2012).

Due to the upper thoracic location of sympathetic preganglionic neurons supplying the heart and upper body vasculature, the segmental level and lesion severity greatly influences the degree of dysfunction following SCI, with lesions above T5 having the most dire CV consequences. Thus, the majority of investigations into CV dysfunction use animal models of complete transection of the upper thoracic spinal cord. These endeavors have traditionally been preferred over contusion models because of the reduced animal to animal variability and the persistent decline in primary cardiac indices that mimics the most severe of the SCI human population. However, these models may not be ideal given that the majority of clinical injuries are incomplete, leading to a wide array of locomotor and CV phenotypes (University of Alabama Birmingham Spinal Cord Injury Model Systems Information Network, https://www.nscisc.uab.edu/). Further, many individuals with lesions at lower thoracic levels also suffer from debilitating bouts of OH, resting tachycardia, and cardiac insufficiency during exercise (Van Loan et al. 1987; Krassioukov and Claydon 2006; Weaver et al. 2012).

Additionally, SCI forces individuals abruptly into a period of very low physical activity, and many are truly sedentary for weeks or months postinjury. This inactivity results in substantial physical deconditioning (Nash 2005) and maladaptive vascular remodeling below the lesion (Boot et al. 2002; de Groot et al. 2006; Thijssen et al. 2012). Many studies have shown that exercise training following SCI can favorably influence hemodynamic, cardiac, and vascular function in rodents and in individuals (West et al. 2014, 2016). (Thijssen et al. 2005, 2006; Harkema et al. 2008). Furthermore, it is generally assumed that initiating exercise rehabilitation acutely following injury is most beneficial to reducing chronic CV dysfunction and impeding the progression of cardiac and vascular because it takes advantage of the inherent plasticity within the central nervous system including the spinal cord (West et al. 2014; West et al. 2015). However, little is known about *how* a newly injured system responds to a bout of exercise or how that response changes over time postinjury. Thus, the primary purpose of this study was to evaluate the hemodynamic response to single bouts of exercise, an “exercise challenge” delivered weekly, in rats with the commonly used low thoracic (T10) contusive injury, but also following a less common, but more clinically relevant high thoracic (T3) contusive injury (DeVivo and Chen 2011). Using implantable telemetry devices, the BP and HR responses to single bouts of swimming exercise delivered weekly were analyzed preinjury and for 10 weeks following SCI. Cardiac structure and function were assessed over time using echocardiography. Dobutamine infusion was employed to test cardiac performance during increased sympathetic activation, irrespective of sympathetic support from damaged spinal autonomic pathways (Plante et al. 2005). We hypothesized that incomplete T3 injuries would result in more severe and longer‐lasting disruptions of CV control as compared to injuries at T10 due to the direct loss of high‐thoracic sympathetic circuitry. However, improvements in hindlimb function and the subsequent increases in spontaneous activity typical of our injury models would reverse the effects of deconditioning and lead to improved CV responses to single bouts of exercise applied at chronic postinjury time points regardless of injury level.

## Methods

### Ethical approval

All animal care and surgical procedures were performed in accordance with the NIH Guidelines and with the approval of the University of Louisville Institutional Animal Care and Use Committee.

### Experimental design

Experiments were conducted on adult female Sprague Dawley rats (250–300 g; Harlan Laboratories, Indianapolis, IN, USA). Prior to SCI injury, animals (*n* = 16) were implanted with telemetry devices to deliver measurements of arterial pressure and ECG (Data Sciences International^®^, St. Paul, MN; HD‐S11 transmitters). Following recovery from device placement, all animals were subjected to bouts of swimming as the exercise challenge over a period of 3 weeks during which hemodynamic responses were quantified. In addition, half of the animals were assessed for cardiac structure and function using echocardiography and dobutamine stress echo (*n* = 8). Prior to SCI, animals were divided into two groups, each of which included four animals that received preinjury echocardiography: T3 SCI (*n* = 8) or T10 SCI (*n* = 8). Rats were then subjected to a moderately severe contusion injury at the T3 or T10 spinal level using the NYU Impactor (Mascis, Rutgers University). All animals were assessed for hemodynamic response to the exercise challenge weekly for 10 weeks (*n* = 8 each group). An additional set of age‐matched animals were used as noninstrumented, noninjured controls (CON, *n* = 8) for cardiac histology.

### Telemetry implantation

All animals were instrumented with HD‐S11 transmitter devices (Data Sciences^®^ International, St. Paul, MN) for in vivo measurement of arterial pressure and ECG as described previously (Brockway et al. 1991). Briefly, under isoflurane anesthesia (2% in oxygen), a ventral midline incision was made in the skin and abdominal wall. The body of the transmitter was placed within the peritoneal cavity and sutured to the abdominal wall musculature. The BP‐sensing cannula was inserted into the abdominal aorta slightly above the bifurcation of the iliac arteries and advanced rostrally to the point where the left renal vein courses over the aorta. The catheter was fixed in place using a small amount of VetBond tissue adhesive (3M™ Vetbond™ Tissue Adhesive, St. Paul, MN). The two biopotential leads were subcutaneously sutured in place under the 12th left rib and over the right pectoralis major muscle for ECG signal recordings in a Modified Lead II configuration (Data Sciences^®^ International *public technical notes in webpage*). The abdominal wall musculature and skin were closed in layers using 4‐0 nylon and 4‐0 silk sutures, respectively. Postoperative care included daily injections of gentamicin sulfate for 7 days (20 mg/kg, S.C.), twice‐daily injections of buprenorphine for 3 days (0.03 mg/kg, S.C., and as needed for pain management thereafter), and twice‐daily 5 mL boluses of lactated ringers for 3 days (and as needed for hydration thereafter). Animals were allowed to recover for 7–10 days following device placement, after which preinjury recordings of arterial pressure and ECG were collected at rest and during the exercise challenge. In the event that a rat showed signs of peritonitis due to the transmitter implantation, daily doses of the nonsteroidal anti‐inflammatory ketoprofen (5 mg/kg S.C.) and additional gentamicin sulfate were administered until symptoms resolved.

### Exercise challenge recording protocol

Following recovery from implantation, rats were reintroduced to the swimming pool and testing conditions. Swimming has been used as both rehabilitation exercise and as an assessment for locomotor recovery following SCI in rodents (Smith et al. 2006b; Gonzenbach et al. 2012). For the purposes of this study, swimming was used as a once‐weekly exercise to challenge the cardiopulmonary system and assess CV control after SCI. Briefly, swim assessments consisted of a 4‐minute session in which the rat swims multiple lengths of a 5‐ft long plexiglass pool. They are repeatedly placed at one end and encouraged to swim to the opposite end where they exit via a padded ramp. Water temperatures are maintained at 33–35°C. This gives the rats incentive to exit the pool but also avoids problems associated with drops in core body temperature and spasticity after injury. Uninjured rats can easily swim upwards of 45 laps in a typical exercise session. Beat‐by‐beat arterial pressure was collected at rest and in response to the exercise challenge at 1000 Hz. In‐cage recordings of arterial pressure and ECG were acquired before swimming (4 min) and during exercise recovery (6 min). Baseline measurements were made three times per week for 3 weeks.

### Spinal cord injury

Approximately 5 weeks after telemetry device placement, rats were given moderately severe contusion injuries using the NYU Impactor. Each animal was anesthetized with a Ketamine (50 mg/kg)/Xylazine (0.024 mg/kg)/Acepromazine (0.005 mg/kg) cocktail (I.P.) and given glycopyrrolate (0.08 mg/kg, IM) prior to the contusion procedure. A dorsal midline incision was made in the superficial muscle overlying either the T1–T4 (T3 contusion) or T7–T12 (T10 contusion) vertebrae. For T3 contusions, a single level laminectomy was made at the T2 vertebral level and using clamps applied to the T1 and T4 spinous processes, the spine was immobilized and positioned for impact. For T10 contusions, the laminectomy was made at the T9 vertebral level and the spine was immobilized using clamps applied to the T8 and T10 spinous processes. The NYU impactor was then used to deliver a moderately‐severe (25 g‐cm) weight drop contusion injury. The muscle and skin overlying the injury was sutured in layers and antibiotic ointment was applied to the incision. Injured animals were monitored on heating pads until they recovered from the anesthesia. Rats were then doubly housed in standard cages with ALPHA‐dri^®^ bedding (Shepherd's™ Specialty Paper, Milford, New Jersey) for the remainder of the study. Postoperative care consisted of daily injections of gentamicin sulfate for 7 days (20 mg/kg, S.C.), twice‐daily injections of buprenorphine for 3 days (0.03 mg/kg, S.C., and as needed for pain management thereafter), and twice‐daily 5 ml boluses of lactated ringers for 3 days (and as needed for hydration thereafter). Manual bladder expression was conducted three times a day until reflexive voiding was reestablished. Rats were maintained on a 12‐h day/night light cycle and had access to standard rat chow and water ad libitum.

### Analysis and behavioral assessments

Cardiovascular data were collected using the PONEMAH^®^ 5.0 software package and DataQuest Acquisition hardware (Data Sciences^®^ International, St. Paul, MN). Initial arterial pressure and pressure‐derived HR data analyses were performed in LabChart version 8.0 (ADInstruments, Colorado Springs, CO). Mean blood pressure (MBP) measurements were calculated for analysis during in cage rest, exercise challenge, and exercise recovery. Mean blood pressure Excursion and HR Excursion during swimming were assessed using a custom Excel macro. Briefly, HR Excursion was determined by calculating the difference in the mean HR from the first to the last 15 sec of the entire four‐minute swim session. Mean blood pressure Excursion was assessed on a lap‐by‐lap basis and calculated as the average difference between the peak and trough values for each lap. To do this, we used the telemetry software to time stamp when the animal was placed into the water and when the animal exited the pool via the ramp. MBP Excursion is the difference between the highest and lowest MBP value during each lap (and then those values are averaged to yield the excursion for the entire 4‐min swim session).

The Louisville Swim Scale (LSS)(Smith et al. 2006a) and the Basso, Beattie, and Bresnahan open field assessment for hindlimb function (BBB)(Basso et al. 1995) were performed weekly to track locomotor recovery. Briefly, the LSS is a testing technique developed by our laboratory to evaluate various characteristics of swimming behavior that are highly compromised following spinal cord contusion: hindlimb movement (i.e., kicking), forelimb dependency, and trunk stability/support. Uninjured rats rely solely on alternating hindlimb kicking for forward propulsion and the forelimbs are only used occasionally for steering purposes. Following moderate‐severe contusion injuries, rats rely on their forelimbs for forward movement and have great difficulty stabilizing their trunks at the water's surface.

### In vivo cardiac echocardiography and dobutamine stress testing

Echocardiographic assessments were performed, using the VisualSonics Vevo^®^ 3100 and MX250S (24 MHz) transducer. Rats were anesthetized with isoflurane (1.75% in oxygen), the thorax was shaved, and the animal was placed in dorsal recumbency. Body temperature, HR, and arterial BP were monitored using the telemetry system as described above. Prior to Dobutamine infusion, standard measures of left ventricular (Mallek et al. 2012) structure (i.e., left ventricular internal diameter during systole and diastole, LVIDs and LVIDd, respectively) and function (i.e., stroke volume, SV, cardiac output, CO, and ejection fraction, EF) were obtained using M‐mode echocardiography along the parasternal short‐axis (SAX) at the mid‐ventricular level. End‐diastolic and end‐systolic volumes were calculated using the Teichholz method. Pulse wave Doppler echocardiography during the apical four‐chamber view was used to estimate early (E) diastolic filling capacity. Following this, the transducer was secured in a stereotaxic stand (VisualSonics) to minimize variation in image capture during the Dobutamine infusion protocol. Dobutamine is a sympathomimetric drug and has been used previously following SCI to increase the chronotrophy and inotrophy of the heart (DeVeau et al. 2017, 2018; Squair et al. 2018). Dobutamine was infused via a tail vein cannula in a step‐wise manner at progressively increasing doses (5, 10, 20, and 30 *μ*g/kg/min) using an automated perfusion pump (KD Scientific, Holliston, MA). Each dose was infused for four minutes to elicit a maximal drug response (Plante et al. 2005), after which an image was captured along the SAX before advancing to the next dose. Results from five cardiac cycles during expiration were averaged for each dose response offline using the VEVO^®^ LAB software. Data were used for between group and time postinjury comparisons. Blood pressure (mean, systolic and diastolic) and HR measures for the final minute of each infusion dose were analyzed, using LabChart version 8.0 as described above (ADInstruments, Colorado Springs, CO).

### Study termination and histological analysis

Upon completion of the study, rats were euthanized with an overdose of sodium pentobarbital (50 mg/kg I.P.), transcardially perfused with phosphate buffer, and fresh dissected to remove the heart and spinal cord. Tissue was postfixed with 4% paraformaldehyde and cryopreserved in 30% sucrose. Spinal cord tissue was sectioned at 30 *μ*m in six sets and assessed for white matter sparing in and around the epicenter (Magnuson et al. 2005; Smith et al. 2006b). Mid‐ventricular heart tissue was sectioned at 10 *μ*m and processed for collagen deposition with Masson's trichrome stain. Images of the left ventricular free wall were captured at 20× magnification. Analysis was completed from five separate sections at least 70 *μ* apart using consistent camera settings. Collagen deposition was calculated as a percent of the total area of the image and percentages from each section were averaged to deliver one value per animal (Radovits et al. 2013).

### Statistical analysis

Behavioral assessments (BBB and LSS) and CV parameters during in‐cage rest and the swimming exercise challenge were analyzed using mixed model analysis of variance (ANOVA) for time with the group factor (T3 and T10). Post hoc t‐tests were completed with Bonferroni. Following normalization to femur length (Lee et al. 2010; Krishnan et al. 2016), echocardiography data were analyzed using repeated measures analysis of variance (RM ANOVA) for group differences and comparisons in dose responses across time. Post hoc t‐tests were completed with Tukey. Terminal histological analyses were analyzed with Independent t‐tests between means with equal or unequal variance, as appropriate, followed by Bonferroni correction for multiple comparisons. Statistical analyses were performed with SPSS (v22, Chicago, IL). All data are shown as mean ± SD and significance was set at *P* ≤ 0.05.

## Results

### Moderately severe contusion results in substantial tissue damage in and around the injury epicenter

**Figure 1:**
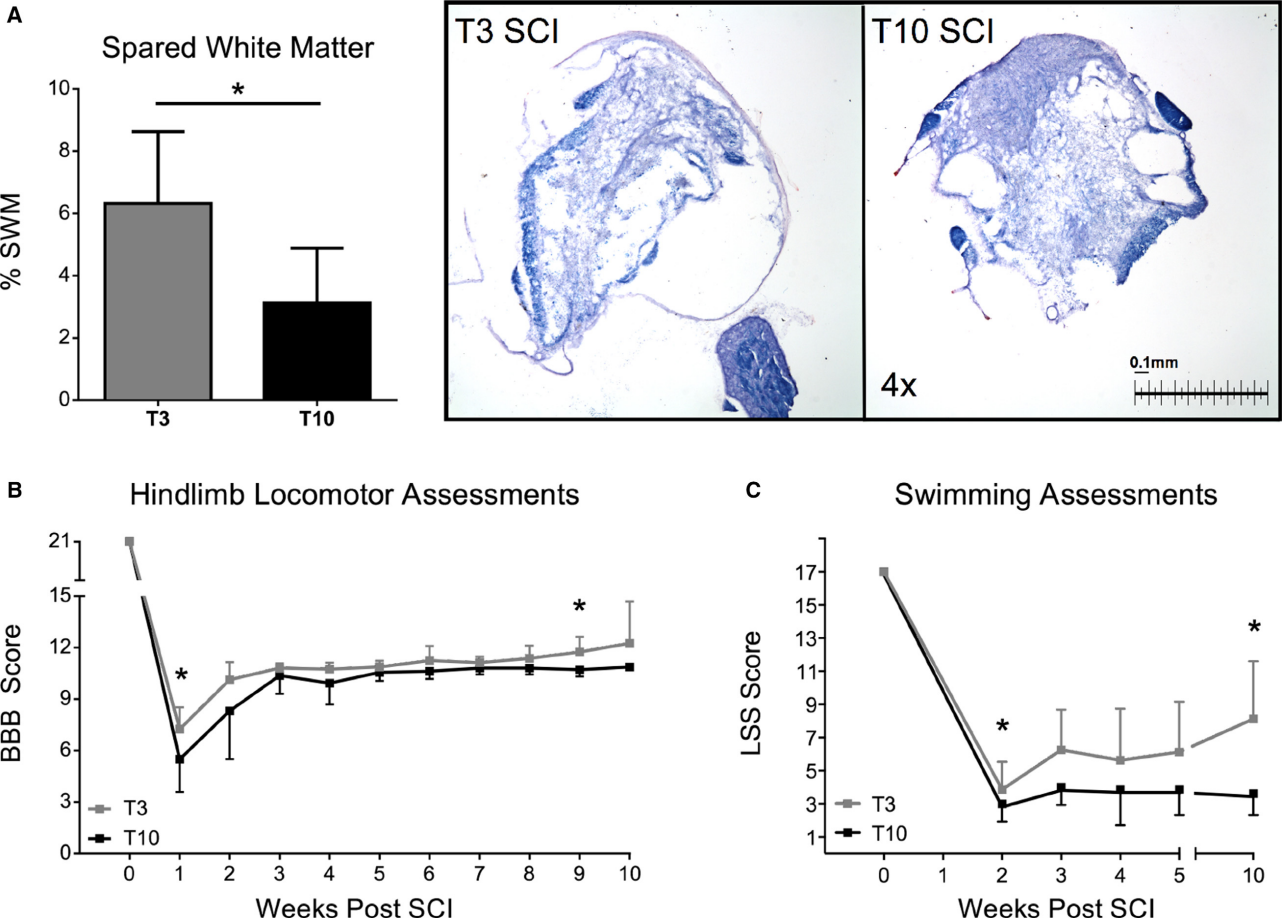
Incomplete contusion results in severity dependent tissue damage and locomotor deficits. (A) Percent spared white matter at the injury epicenter following T3 and T10 contusion. (B) Group comparisons of weekly BBB scores over time in T3 and T10 animals. Significant group differences were noted at weeks one and nine postinjury. (C) Group comparisons of weekly performance during swimming assessments. Significant group differences are noted at three and ten weeks post‐SCI. Statistical significance was assessed using Mixed Model ANOVA with Bonferroni *post hoc* t‐test. Data are displayed as mean ± SD (*n* = 8 each group) and statistical significance was set as * *P* ≤ 0.05.

Following T3 contusion, the average area of spared white matter (SWM), assessed as darkly stained compact white matter, was 0.1581 mm^2^ and the percent of SWM ranged from 3.40 to 9.52 percent overall (group average 6.325 ± 2.3 percent). Contusion to the lower thoracic cord (T10) also resulted in substantial white matter damage, as the average area of sparing was 0.0781 mm^2^. The percent of spared white matter ranged from approximately 0.88 to 6.44 percent, and averaged 3.125 percent overall. Group comparisons of percent SWM are represented in Figure 1A.

Locomotor assessments postinjury were executed at the beginning of each week prior to the swimming exercise challenge (Fig. 1B). Group comparisons revealed similar patterns of hindlimb recovery in T3 and T10 animals, with T3 rodents having slightly higher mean group BBB scores at weeks 1 and 9 postinjury (*P* ≤ 0.05). These differences are likely due to partial sparing of abdominal and trunk muscle innervation in our model of incomplete T3 injury. Time‐wise comparisons showed that in both groups, BBB scores measured during week 1 were significantly lower than those measured at subacute (weeks 2–5) and chronic (week 10) time points (data not shown; T3 and T10: week 1 vs. weeks 3–10, *P* ≤ 0.001). Most animals only regained frequent, but not consistent, weight‐supported hindlimb stepping and toe clearance also remained poor for the duration of the study.

Swimming behavior was evaluated weekly starting 2 weeks after injury (Fig. 1C). LSS assessments revealed a severe drop in swimming ability postinjury with no improvements over time in either T3 or T10 groups. Group comparisons revealed slightly better performance on the LSS in T3 animals at weeks 3 and 10 postinjury. As shown by the mean LSS score of 3.5, T10 animals relied solely on their forelimbs for forward propulsion and had great difficulty stabilizing their trunk during swimming for the entirety of the study. Conversely, animals with T3 contusions occasionally used alternating hindlimb kicks to help propel themselves across the pool. This disparity likely relates to the fact that T10 contusions damage areas of the spinal cord responsible for the control of abdominal and trunk musculature (T7‐T11). Without adequate trunk control, animals are unable to properly recruit the hindlimbs to participate in swimming exercises.

### Chronic CV function at rest is not compromised by moderately‐severe contusion of the thoracic spinal cord

Weekly assessments of resting MBP and HR were completed in T3 and T10 contusion groups. No differences were noted in MBP between groups prior to contusion injury (*P* = 1.0). Resting HR was slightly higher in T10 animals prior to injury and 2 weeks postcontusion as compared to T3 (*P* ≤ 0.05). Group differences in resting CV parameters following contusion are highlighted in Table 1.

**Table 1:** Group comparisons of average blood pressure and heart rate over time in T3 and T10 animals

	Preinjury	Week 1	Week 2	Week 3	Week 4	Week 5	Week 10
Hemodynamics during in cage rest							
* MBP (mmHg)*							
T3	120.92 ± 4.62	119.37 ± 5.75	122.69 ± 5.91	123.20 ± 5.13	122.93 ± 5.39	125.65 ± 5.48	122.10 ± 2.70
T10	120.74 ± 3.69	120.46 ± 3.53	123.61 ± 2.63	125.43 ± 7.00	126.00 ± 3.80	124.31 ± 3.15	121.38 ± 4.98
* HR (bpm)*							
T3	422.84 ± 20.16 †	465.74 ± 22.50	410.12 ± 32.95 †	429.58 ± 40.30	443.98 ± 21.34	434.51 ± 17.51	442.58 ± 15.03
T10	445.97 ± 19.48	474.47 ± 29.14	440.87 ± 21.12	442.71 ± 21.80	442.72 ± 24.63	441.83 ± 29.19	426.10 ± 27.73
Hemodynamics during swimming exercise challenge							
* MBP (mmHg)*							
T3	129.45 ± 1.62	114.25 ± 3.03 ††	126.75 ± 1.78 †	130.24 ± 1.82 †	133.57 ± 1.58	134.77 ± 2.06	135.13 ± 1.92
T10	127.02 ± 4.14	122.72 ± 1.97	131.82 ± 3.96	135.77 ± 5.75	136.30 ± 4.06	136.00 ± 5.84	133.98 ± 6.15
* HR (bpm)*							
T3	478.97 ± 5.15	475.02 ± 10.99	469.43 ± 7.03	475.28 ± 7.90	486.90 ± 6.49	487.39 ± 7.78	475.64 ± 4.97
T10	484.88 ± 14.54	472.58 ± 19.89	480.52 ± 17.75	475.10 ± 16.04	476.45 ± 16.29	480.67 ± 9.84	464.13 ± 19.14
Hemodynamics during exercise recovery							
* MBP (mmHg)*							
T3	127.23 ± 2.94	112.96 ± 3.17	115.36 ± 7.89 †	118.67 ± 7.08	120.73 ± 5.25	121.95 ± 3.75	123.48 ± 4.68
T10	124.75 ± 3.01	112.74 ± 1.76	123.70 ± 3.00	124.78 ± 6.20	124.70 ± 4.26	124.89 ± 2.74	122.53 ± 5.93
* HR (bpm)*							
T3	480.75 ± 15.47	460.65 ± 11.95	463.56 ± 25.23	474.61 ± 18.47	487.93 ± 24.32	488.48 ± 16.79	477.92 ± 20.99
T10	484.90 ± 17.07	459.00 ± 18.02	487.67 ± 13.06	477.80 ± 17.80	479.22 ± 21.76	483.29 ± 11.43	458.07 ± 34.51

Statistical significance was assessed using Mixed Model ANOVA with Bonferroni *post hoc* t‐test. Data are displayed as mean ± SD (*n* = 8 each group except week 1, *n* = 4 each group) and statistical significance was set as ^†^ *P* ≤ 0.05 and ^††^ *P* ≤ 0.01.

Moderately severe contusion of the lower thoracic spinal cord did not alter resting CV hemodynamics over time (T10, Table 1). Resting MBP and HR were similar to preinjury measurements at all time points assessed. Conversely, T3 animals experienced resting tachycardia 1 week following contusion, that quickly resolved by 2 weeks postinjury (Table 1; Week 1 vs. Preinjury, *P* = 0.017; Week 1 vs. Week 2, *P* = 0.02). Mean blood pressure in T3 animals at rest was within normal ranges for all time points assessed postinjury (Table 1).

### Hemodynamic responses to the exercise challenge are disrupted after high and low thoracic contusion

CV responses to an exercise challenge (swimming) were evaluated weekly before and after thoracic contusion injury. During preinjury assessments, there were modest pressor responses of 6 and 7.5% (T3 and T10 respectively) at the initiation of swimming that was maintained throughout the 4‐minute session. The heart rat also showed increases (9 and 13% respectively) and remained quite stable for the duration of the exercise challenge (Table 1). One week following contusion, T3 and T10 animals were still able to mount a modest pressor response during swimming, although it was somewhat blunted following T3 contusion (Figure 4B; T3: Week 1 vs. Preinjury and Weeks 3, 4, 5, and 10, *P* ≤ 0.01). As illustrated in Table 1, animals with T10 contusion injuries were able to achieve greater pressor responses to the exercise challenge when compared to T3 animals in the acute and subacute time points postinjury (Table 1; Weeks 1, 2, and 3, *P* ≤ 0.05).

**Figure 2:**
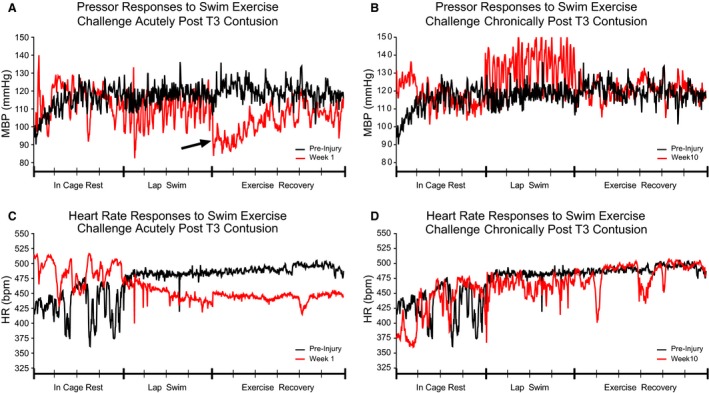
Rodents with high thoracic contusion are unable to maintain cardiovascular control during swimming exercise challenge. Representative MBP (A) and HR (C) responses to swimming exercise challenge before (black lines) and one week post‐T3 moderate contusion (red lines). Note the postexertional hypotension during the Exercise Recovery period acutely after injury (A, arrow). Representative MBP (B) and HR (D) responses to swimming exercise challenge before (black lines) and 10 weeks post‐T3 moderate contusion (red lines). Note the elevated pressor response to swim challenge at ten weeks post‐SCI. Data have been down sampled from 1000 Hz to display one data point per second. Individual recordings of In Cage Rest (4 min), Lap Swim (4 min), and Exercise Recovery (6 min) are displayed as one continuous MBP or HR trace. Each tick on the *x*‐axis represents one minute.

**Figure 3:**
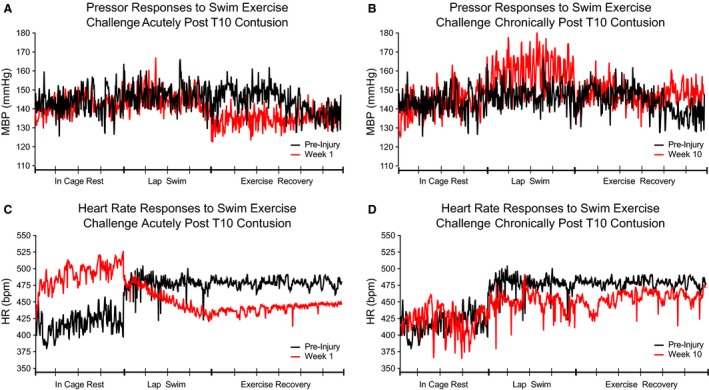
Rodents with low thoracic contusion are unable to maintain cardiovascular control during swimming exercise challenge. Representative MBP (A) and HR (C) responses to swimming exercise challenge before (black lines) and one week post‐T10 moderate contusion (red lines). Note the drastic fall in HR from the beginning to the end of the four minute swimming exercise challenge. Representative MBP (B) and HR (D) responses to swimming exercise challenge before (black lines) and 10 weeks post‐T10 moderate contusion (red lines). Note the elevated pressor response to swim challenge at ten weeks post‐SCI. Data have been down sampled from 1000 Hz to display one data point per second. Individual recordings of In Cage Rest (4 min), Lap Swim (4 min), and Exercise Recovery (6 min) are displayed as one continuous MBP or HR trace. Each tick on the *x*‐axis represents one minute.

**Figure 4:**
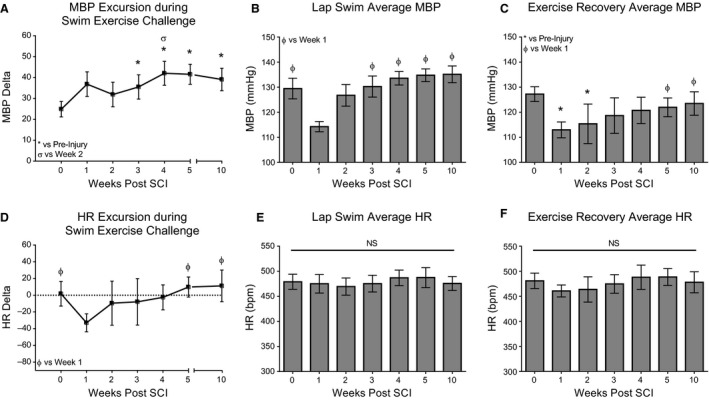
Lack of cardiovascular control during exercise challenge persists for many weeks following moderate T3 contusion. (A) Average MBP Excursion measured prior to SCI and each week following injury. Oscillatory changes in MBP during each swim lap are averaged for each time point. The inability to maintain MBP during exercise challenge increased with time postinjury. (B) Average MBP during the four‐minute swim session. Note the exertional hypotension one week after injury. (C) Average MBP during the Exercise Recovery period. (D) HR Excursion during the four‐minute swim session. Note the drastic drop in HR from the beginning to the end of swimming acutely after injury. (E) Average HR during the four‐minute swim session. (F) Average HR during the Exercise Recovery period. Statistical significance was assessed using Mixed Model ANOVA with Bonferroni *post hoc* t‐test. Data are displayed as mean ± SD (*n* = 4 for week 1, *n* = 8 for all other time points) and statistical significance was set as * *P* ≤ 0.05 vs. preinjury, ^*ϕ*^ *P* ≤ 0.05 vs. week 1, and ^Ϭ^ *P* ≤ 0.05 vs. week 2.

**Figure 5:**
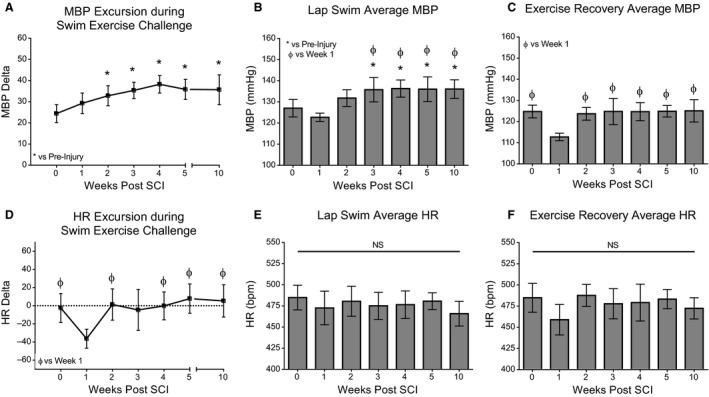
The inability to regulate blood pressure control in response to exercise challenge persists for many weeks following T10 contusion. (A) Average MBP Excursion measured prior to SCI and each week following injury. Oscillatory changes in MBP during each swim lap are averaged for each time point. The inability to maintain MBP during exercise challenge increased with time postinjury. (B) Average MBP during the four‐minute swim session. Note the increased pressor response chronically after injury. (C) Average MBP during the Exercise Recovery period. Note the postexertional hypotension one week after injury. (D) HR Excursion during the four‐minute swim session. Note the drastic drop in HR from the beginning to the end of swimming acutely after injury. (E) Average HR during the four‐minute swim session. (F) Average HR during the Exercise Recovery period. Statistical significance was assessed using Mixed Model ANOVA with Bonferroni *post hoc* t‐test. Data are displayed as mean ± SD (*n* = 4 for week 1, *n* = 8 for all other time points) and statistical significance was set as * *P* ≤ 0.05 vs. preinjury and ^*ϕ*^ *P* ≤ 0.05 vs. week 1.

Acutely following both T3 and T10 contusion, the HR dropped considerably as the swimming session progressed and remained low for at least several minutes after the exercise challenge had ceased. Bradycardia during the swimming exercise, measured as HR Excursion, is significantly greater in magnitude at 1 week verses almost all later time points after both T3 and T10 contusions (Fig. 4D; T3: Week 1 vs. Preinjury and Weeks 5 and 10, *P* ≤ 0.05. Fig. 5D; T10: Week 1 vs. Preinjury and Weeks 2 and 4, *P* ≤ 0.05; Week 1 vs. Weeks 5 and 10, *P* ≤ 0.01). Figures 2 and 3 illustrate representative recordings of MBP and HR during in‐cage rest, swimming exercise challenge, and exercise recovery in individual rodents at baseline (shown in black) and acutely post‐T3 (Fig. 2A, C) or T10 (Fig. 3A, C) contusion (shown in red).

Deficits in CV control during the exercise challenge persisted for many weeks following injury. Lack of BP control intensified with time postinjury, as the range in MBP values measured on a lap‐by‐lap basis increased substantially in amplitude until week 10. Visually, this is shown as large saw‐tooth patterns during bouts of swimming exercise challenge in representative hemodynamic traces at sub‐acute and chronic time points (Fig. 2B and 3B) and is quantified as MBP Excursion (Fig. 4A, T3: Preinjury vs. Weeks 3, 4, 5, and 10, *P* ≤ 0.01. Fig. 5A, T10: Preinjury vs. Week 2, *P* ≤ 0.05; Preinjury vs. Weeks 3‐5 and 10, *P* ≤ 0.01). Furthermore, the average MBP in T10 animals during the four‐minute exercise challenge was significantly greater during sub‐acute and chronic postinjury assessments than at preinjury and week 1 (Fig. 5B; Preinjury vs. Weeks 3–5 and 10, *P* ≤ 0.05; Week 1 vs Weeks 3–5 and 10, *P* ≤ 0.05). Average HR varied considerably and measurements were not significantly different during the exercise challenge over time or between groups (Fig. 4E and 5E).

### Contused animals are unable to maintain blood pressure control during the exercise recovery period acutely after injury

Following the cessation of the swimming exercise challenge, rodents with thoracic contusion injuries experienced short bouts of exertional hypotension during the exercise recovery period at one and 2 weeks following T3 contusion (Fig. 2A, arrows; Fig. 4C, Week 1 vs. Preinjury and Week 2, *P* ≤ 0.01; and Week 1 vs Weeks 5 and 10, *P* ≤ 0.01), and at 1 week following T10 contusion (Fig. 5C; Week 1 vs. Preinjury and Weeks 2, 3, 5, and 10, *P* ≤ 0.01). Group differences in hemodynamic control during the exercise recovery period were minimal, with T10 animals showing slightly greater MBP at 2 weeks postinjury (Table 1, *P* = 0.014). Average HR varied considerably and measurements were not significantly different during the exercise recovery period over time or between groups (Fig. 4F and 5F).

### Echocardiography

Group comparisons of echocardiogram data revealed few differences in cardiac structure and function following T3 and T10 injuries. No differences were noted in T3 body weight, heart mass, or collagen deposition verses uninjured, age‐matched controls (Table 2); however, the difference in the body mass to heart mass ratio between T3 and uninjured control animals approached significance (*P* = 0.052). Heart mass and the ratio between heart and body mass were significantly higher in T10 contused animals verses uninjured, age‐matched controls (Table 2; *P* ≤ 0.01).

**Table 2:** Anatomical and echocardiographic data from moderately‐contused rodents and uninjured controls

	T3 SCI	T10 SCI	Un‐injured Control	Key
Anatomical data				
Body mass	290.13 ± 13.01	304.25 ± 25.63	298.88 ± 18.06	*Dose vs. Dose 0 *μ*g
Heart mass	1.0837 ± 0.1097	1.1797 ± 0.0559 τ	1.0022 ± 0.0963	†Dose vs. Dose 5 *μ*g
Heart/Body mass ratio	0.0037 ± 0.0003	0.0039 ± 0.0002 τ	0.0034 ± 0.0004	ǂDose vs. Dose 10 *μ*g
Area collagen	0.0031 ± 0.0015	0.0019 ± 0.0011	0.0031 ± 0.0006	τSCI Group vs. Control
Percent collagen	0.8400 ± 0.4000	0.5153 ± 0.2985	0.8264 ± 0.1572	^*ϕ*^T3 vs T10 SCI Groups

T3 Echocardiographic Data during dobutamine stress testing					
	*Dose*	Preinjury	Week 1	Week 5	Week 10
Dimensions					
LVIDd (mm)	0 *μ*g	7.48 ± 0.19	6.86 ± 0.66	6.58 ± 0.82	6.87 ± 0.63
	5 *μ*g	7.60 ± 0.33	7.30 ± 0.52	6.88 ± 0.69	6.53 ± 1.04
	10 *μ*g	7.26 ± 0.26	7.16 ± 0.68	6.63 ± 0.58	6.69 ± 0.60
	20 *μ*g	7.06 ± 0.18 †	7.16 ± 0.55	6.61 ± 0.39	6.74 ± 0.46
	30 *μ*g	7.00 ± 0.26 *, †	6.94 ± 0.53	6.51 ± 0.44	6.61 ± 0.56
LVIDs (mm)	0 *μ*g	4.28 ± 0.57	4.34 ± 0.52	3.87 ± 0.73	3.42 ± 0.84
	5 *μ*g	4.34 ± 0.72	3.79 ± 0.37	3.41 ± 1.29	2.68 ± 0.57
	10 *μ*g	2.88 ± 0.68 *, †	2.87 ± 0.54 *	2.68 ± 0.87 *	2.21 ± 0.54 *
	20 *μ*g	2.18 ± 0.50 *, †	2.55 ± 0.80 *, †	2.51 ± 1.86 *	2.01 ± 0.56 *
	30 *μ*g	2.17 ± 0.65 *, †	2.13 ± 0.70 *, †	1.75 ± 0.81 *, †	1.61 ± 0.48 *, †
EDV (*μ*L)	0 *μ*g	297.04 ± 17.44	246.10 ± 53.67	225.01 ± 60.60	246.40 ± 51.05
	5 *μ*g	307.35 ± 29.81	282.22 ± 44.57	247.86 ± 54.61	223.28 ± 76.00
	10 *μ*g	277.89 ± 22.55	270.61 ± 57.04	227.44 ± 43.72	232.17 ± 46.20
	20 *μ*g	260.84 ± 15.36 †	269.92 ± 46.41	225.32 ± 30.09	235.36 ± 35.84
	30 *μ*g	256.02 ± 21.41 *, †	251.59 ± 41.86	217.28 ± 32.62	225.64 ± 42.46
ESV (*μ*L)	0 *μ*g	83.52 ± 26.21	85.91 ± 23.55	67.05 ± 30.93	51.27 ± 30.76
	5 *μ*g	86.98 ± 31.12 ^*ϕ*^	62.17 ± 13.93	54.92 ± 49.72	27.98 ± 12.92
	10 *μ*g	33.57 ± 17.97 *, †	32.68 ± 13.47 *	29.57 ± 21.82	17.59 ± 9.15
	20 *μ*g	16.82 ± 8.73 *, †	26.12 ± 16.93 *	36.90 ± 54.53	14.10 ± 8.70
	30 *μ*g	17.38 ± 10.84 *, †	16.81 ± 11.21 *, †	11.56 ± 12.85 *, †	8.19 ± 6.35 *
Systolic Function					
SV (*μ*L)	0 *μ*g	213.52 ± 30.51	160.18 ± 31.14	157.97 ± 45.71	195.14 ± 24.15
	5 *μ*g	220.37 ± 7.16 *	220.05 ± 35.29	192.94 ± 35.19	195.30 ± 77.31
	10 *μ*g	244.32 ± 15.78 *	237.94 ± 46.22	197.88 ± 39.36	214.58 ± 47.15
	20 *μ*g	244.02 ± 8.50 *	243.80 ± 38.39	188.42 ± 27.71	221.26 ± 33.75
	30 *μ*g	238.63 ± 10.74 *	234.78 ± 32.61	205.72 ± 24.90 *	217.45 ± 38.45
EF (%)	0 *μ*g	71.85 ± 9.09	65.37 ± 3.14	70.32 ± 8.92	80.19 ± 7.90
	5 *μ*g	72.19 ± 7.97	78.03 ± 3.36	79.42 ± 15.17	85.97 ± 9.08
	10 *μ*g	88.13 ± 5.98 *, †	88.22 ± 3.51 *	87.15 ± 8.04 *	92.15 ± 4.57
	20 *μ*g	93.65 ± 3.02 *, †	90.64 ± 5.91 *	85.38 ± 20.81 *	94.02 ± 3.36 *
	30 *μ*g	93.42 ± 3.79 *, †	93.67 ± 3.87 *, †	95.02 ± 4.99 *, †	96.51 ± 2.18 *
CO (mL·min^−1^)	0 *μ*g	69.26 ± 15.91	50.53 ± 6.25	46.55 ± 14.79	58.77 ± 12.77
	5 *μ*g	67.94 ± 4.86	66.40 ± 6.12	55.08 ± 16.02	59.83 ± 28.61
	10 *μ*g	82.90 ± 5.40	76.68 ± 7.61	57.38 ± 18.33	68.32 ± 21.60
	20 *μ*g	87.28 ± 1.58	84.50 ± 10.33 *	60.79 ± 9.28	76.15 ± 11.87
	30 *μ*g	86.09 ± 5.05	84.94 ± 8.27 *	69.01 ± 9.56	78.32 ± 10.81
Diastolic Function					
E (cm·sec^−1^)	0 *μ*g	723.19 ± 131.09	627.70 ± 116.52	690.76 ± 68.30	739.94 ± 67.51
Hemodynamics					
SBP (mmHg)	0 *μ*g	114.32 ± 12.91	89.29 ± 3.43 ^*ϕ*^	87.28 ± 5.31	88.75 ± 9.71
	5 *μ*g	112.79 ± 8.78	105.60 ± 7.80	92.64 ± 11.74	95.83 ± 10.52
	10 *μ*g	113.52 ± 8.64	105.65 ± 4.70	95.29 ± 11.27	104.85 ± 14.58
	20 *μ*g	111.49 ± 8.70	103.27 ± 5.14	97.51 ± 5.61	102.50 ± 14.70
	30 *μ*g	106.13 ± 8.30	98.98 ± 5.17	93.62 ± 7.36	94.20 ± 10.61
DBP (mmHg)	0 *μ*g	73.25 ± 9.83	55.86 ± 2.87 ^*ϕ*^	53.34 ± 4.40 ^*ϕ*^	53.25 ± 5.10
	5 *μ*g	70.73 ± 7.60	55.66 ± 7.96	49.89 ± 7.46	52.57 ± 5.09
	10 *μ*g	66.15 ± 7.70	52.23 ± 5.62	48.94 ± 3.19 ^*ϕ*^	53.31 ± 5.00
	20 *μ*g	64.50 ± 6.78 *	51.54 ± 4.53	47.67 ± 2.20 ^*ϕ*^	52.18 ± 3.55
	30 *μ*g	60.45 ± 6.21 *, †	49.45 ± 5.92 *, †	46.62 ± 2.83 ^*ϕ*^	47.67 ± 3.38 ^*ϕ*^
MBP (mmHg)	0 *μ*g	86.94 ± 9.22	67.01 ± 2.64 ^*ϕ*^	64.66 ± 4.55	65.08 ± 6.02
	5 *μ*g	84.75 ± 4.72	72.31 ± 7.35	64.14 ± 8.65	66.99 ± 6.01
	10 *μ*g	81.94 ± 4.94	70.03 ± 3.83	64.39 ± 5.04	70.48 ± 7.50
	20 *μ*g	80.16 ± 4.43	68.79 ± 3.24	64.28 ± 1.96	68.95 ± 6.75
	30 *μ*g	75.68 ± 3.70	65.96 ± 4.96	62.29 ± 4.14	63.18 ± 4.61
HR (bpm)	0 *μ*g	315.07 ± 29.28	327.55 ± 10.77	304.45 ± 34.31	294.68 ± 31.23
	5 *μ*g	304.79 ± 15.65	310.50 ± 28.22	295.90 ± 40.74	303.17 ± 28.08
	10 *μ*g	334.05 ± 13.94	333.11 ± 32.30	299.89 ± 43.12	316.43 ± 31.25
	20 *μ*g	348.71 ± 13.65	355.37 ± 39.98	328.57 ± 17.43	338.24 ± 22.14
	30 *μ*g	354.24 ± 9.28	374.11 ± 45.38 †	338.15 ± 9.10	357.13 ± 47.34 *

T10 Echocardiographic data during dobutamine stress testing					
	*Dose*	Preinjury	Week 1	Week 5	Week 10
Dimensions					
LVIDd (mm)	0 *μ*g	7.31 ± 0.24	7.26 ± 0.17	7.09 ± 0.47	6.95 ± 0.31
	5 *μ*g	7.09 ± 0.49 *	7.11 ± 0.15	6.88 ± 0.32	6.99 ± 0.19
	10 *μ*g	6.69 ± 0.46 *, †	6.99 ± 0.19	6.66 ± 0.36 *	6.77 ± 0.24
	20 *μ*g	6.56 ± 0.30 *, †	6.99 ± 0.18	6.65 ± 0.29 *	6.84 ± 0.20
	30 *μ*g	6.45 ± 0.53 *, †	6.90 ± 0.15 *	6.61 ± 0.35 *	6.65 ± 0.26 *, †
LVIDs (mm)	0 *μ*g	4.27 ± 0.09	4.17 ± 0.55	4.07 ± 0.38	4.00 ± 0.51
	5 *μ*g	3.13 ± 0.30 *	3.50 ± 0.32 *	3.25 ± 0.40 *	3.26 ± 0.36 *
	10 *μ*g	2.37 ± 0.20 *, †	2.74 ± 0.67 *, †	2.48 ± 0.55 *, †	2.31 ± 0.25 *, †
	20 *μ*g	2.08 ± 0.40 *, †	2.53 ± 0.47 *, †	2.20 ± 0.44 *, †	2.23 ± 0.54 *, †
	30 *μ*g	1.99 ± 0.44 *, †	1.91 ± 0.62 *, †, ǂ	1.97 ± 0.24 *, †	2.00 ± 0.37 *, †
EDV (*μ*L)	0 *μ*g	282.18 ± 20.48	277.58 ± 14.84	264.11 ± 38.12	251.95 ± 25.56
	5 *μ*g	263.95 ± 39.18	264.29 ± 12.73	245.95 ± 24.90	255.18 ± 15.49
	10 *μ*g	231.43 ± 34.16 *	255.16 ± 16.06	229.02 ± 27.11 *	237.42 ± 18.94
	20 *μ*g	220.90 ± 22.85 *, †	254.76 ± 15.29	227.78 ± 21.76 *	242.89 ± 16.10
	30 *μ*g	213.52 ± 39.30 *, †	247.52 ± 12.01	225.27 ± 25.90 *	227.59 ± 20.18
ESV (*μ*L)	0 *μ*g	81.94 ± 4.04	78.90 ± 24.65	73.51 ± 16.03	71.53 ± 23.15
	5 *μ*g	39.10 ± 9.04 *, ^*ϕ*^	51.40 ± 11.62 *	43.30 ± 12.94 *	43.37 ± 11.59 *
	10 *μ*g	19.81 ± 4.22 *, †	30.14 ± 18.44 *, †	23.24 ± 13.96 *	18.67 ± 5.06 *, †
	20 *μ*g	14.73 ± 7.37 *, †	23.96 ± 11.15 *, †	17.16 ± 8.41 *, †	18.17 ± 11.72 *, †
	30 *μ*g	13.47 ± 7.03 *, †	12.94 ± 8.55 *, †	12.42 ± 3.45 *, †	13.38 ± 6.69 *, †
Systolic Function					
SV (*μ*L)	0 *μ*g	200.25 ± 18.84	198.68 ± 24.00	190.60 ± 23.93	180.42 ± 12.47
	5 *μ*g	224.85 ± 32.54	212.89 ± 12.84	202.66 ± 19.68	211.81 ± 19.75 *
	10 *μ*g	211.62 ± 32.00	225.01 ± 7.63	205.78 ± 22.88	218.75 ± 19.66 *
	20 *μ*g	206.17 ± 22.85	230.79 ± 4.44 *	210.62 ± 16.94	224.72 ± 16.85 *
	30 *μ*g	200.05 ± 32.58	234.58 ± 4.33 *	212.85 ± 22.69	214.21 ± 22.92 *
EF (%)	0 *μ*g	70.88 ± 1.93	71.63 ± 8.26	72.36 ± 2.66	71.98 ± 6.29
	5 *μ*g	85.23 ± 2.06 *	80.59 ± 3.95 *	82.51 ± 4.20 *	82.95 ± 4.64 *
	10 *μ*g	91.41 ± 1.42 *	88.45 ± 6.26 *, †	90.05 ± 5.25 *, †	92.10 ± 2.3 *, †
	20 *μ*g	93.34 ± 3.19 *, †	90.76 ± 3.71 *, †	92.62 ± 3.28 *, †	92.57 ± 4.72 *, †
	30 *μ*g	93.97 ± 2.26 *, †	94.89 ± 3.26 *, †, ǂ	94.57 ± 1.07 *, †	94.04 ± 3.26 *, †
CO (mL·min^−1^)	0 *μ*g	62.92 ± 10.11	67.40 ± 6.20	58.09 ± 7.74	53.62 ± 12.85
	5 *μ*g	74.22 ± 17.21	73.97 ± 4.95	63.15 ± 7.00	60.44 ± 8.92
	10 *μ*g	77.11 ± 18.67	82.26 ± 5.86 *	68.00 ± 7.61	67.11 ± 8.10
	20 *μ*g	80.09 ± 15.14 *	88.54 ± 4.02 *	71.25 ± 5.70	69.66 ± 7.07 *
	30 *μ*g	87.19 ± 32.85 *	92.21 ± 6.04 *, †	72.24 ± 1.81	69.05 ± 10.81 *
Diastolic Function					
E (cm·sec^−1^)	0 *μ*g	67.58 ± 22.34	69.73 ± 8.75	77.37 ± 25.57	83.15 ± 13.80
*Hemodynamics*					
SBP (mmHg)	0 *μ*g	105.83 ± 7.59	104.09 ± 8.57 ^*ϕ*^	101.41 ± 14.00	102.97 ± 9.19
	5 *μ*g	109.60 ± 7.26	107.27 ± 7.58	100.86 ± 9.99	104.77 ± 7.72
	10 *μ*g	109.73 ± 8.22	106.45 ± 6.15	99.17 ± 8.32	102.49 ± 7.31
	20 *μ*g	111.63 ± 14.35	103.69 ± 5.67	97.43 ± 7.69	104.15 ± 5.02
	30 *μ*g	108.98 ± 15.56	101.08 ± 5.60	94.63 ± 6.48	99.75 ± 5.52
DBP (mmHg)	0 *μ*g	59.93 ± 14.13	62.05 ± 1.23 ^*ϕ*^	63.16 ± 6.29 ^*ϕ*^	61.08 ± 5.05
	5 *μ*g	64.94 ± 7.17	59.77 ± 2.23	57.92 ± 4.76	56.25 ± 6.44
	10 *μ*g	62.72 ± 7.23	56.98 ± 3.91	54.72 ± 2.39 ^*ϕ*^	52.73 ± 4.01
	20 *μ*g	64.87 ± 11.25	55.08 ± 3.47	53.95 ± 1.83 ^*ϕ*^	54.29 ± 3.81
	30 *μ*g	63.53 ± 11.81	53.99 ± 3.96	52.56 ± 1.40 ^*ϕ*^	52.62 ± 1.21 ^*ϕ*^
MBP (mmHg)	0 *μ*g	75.02 ± 11.12	76.06 ± 2.91 ^*ϕ*^	75.91 ± 8.85	75.04 ± 6.31
	5 *μ*g	79.83 ± 6.99	75.61 ± 1.46	72.23 ± 6.42	72.42 ± 6.79
	10 *μ*g	78.39 ± 7.32	73.47 ± 1.98	69.53 ± 4.21	69.32 ± 4.95
	20 *μ*g	80.46 ± 12.11	71.29 ± 1.52	68.44 ± 3.65	70.91 ± 3.36
	30 *μ*g	78.68 ± 12.88	69.69 ± 2.17	66.59 ± 2.83	68.33 ± 2.12
HR (bpm)	0 *μ*g	311.07 ± 40.96	331.18 ± 32.57	300.48 ± 54.59	285.30 ± 40.48
	5 *μ*g	323.36 ± 39.19	345.64 ± 18.25	309.96 ± 42.18	285.33 ± 35.50
	10 *μ*g	354.44 ± 42.39	362.48 ± 19.28	328.32 ± 40.06	302.67 ± 32.83
	20 *μ*g	383.93 ± 44.49 *, †	380.15 ± 21.98	337.56 ± 35.33	302.55 ± 39.32
	30 *μ*g	393.18 ± 41.64 *, †	391.43 ± 29.84 *	340.28 ± 34.81	321.58 ± 28.04

LVIDd, left ventricular internal diameter during diastole; LVIDs left ventricular internal diameter during systole; EDV, end‐diastolic volume; ESV, end‐systolic volume; SV, stroke volume; EF, ejection fraction; CO, cardiac output; E, transmitral filling velocity during early diastole; SBP, systolic blood pressure; DBP, diastolic blood pressure; MBP, mean blood pressure; HR, heart rate. Data are displayed as mean ± SD (*n* = 4 each group for echocardiographic data; *n* = 8 each group for anatomical data). * *P* ≤ 0.05 dose vs. 0 *μ*g dose; ^†^ *P* ≤ 0.05 dose vs. 5 *μ*g dose; ^ǂ^ *P* ≤ 0.05 dose vs. 10 *μ*g dose; ^τ^ *P* ≤ 0.05 moderate SCI vs. uninjured control group differences; and ^ϕ^ *P* < 0.05, T3 vs. T10 SCI groups.

**Figure 6:**
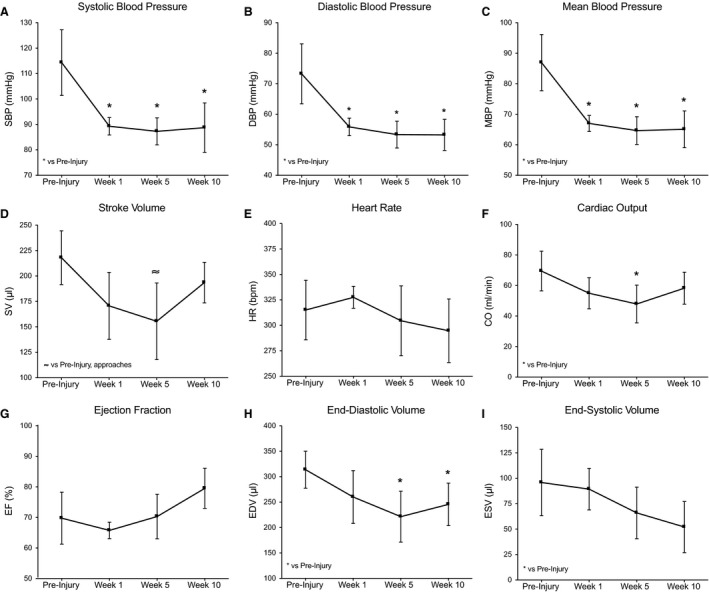
Timewise comparison of cardiac function following T3 moderate contusion using echocardiography. Compared to preinjury, pressor responses in the presence of isoflurane anesthesia were blunted at all time points assessed (SBP (A), DBP (B), and MBP (C)). Measures of systolic function (SV (D), CO (F), and EF (G)) over time indicate a recovery of function at 10 weeks post‐SCI. End‐diastolic volume (H) was significantly lower at chronic time points; ESV (I) and HR (E) were not different from preinjury. Data are displayed as mean ± SD (*n* = 4) and significance is set at * *P* ≤ 0.05 vs. preinjury.

Following T10 contusion, BP measurements during echocardiography were similar to preinjury parameters at all time points assessed (Table 2). Conversely, BP measurements (systolic, diastolic, and mean blood pressure) during echocardiography (i.e., in the presences of isoflurane anesthesia) in T3 animals were significantly reduced postinjury (Fig. 6A–C; Preinjury vs. Weeks 1, 5, and 10, *P* ≤ 0.001). Group comparisons also revealed significant differences in SBP, MBP, and DBP 1 week postcontusion (Table 2, *P* ≤ 0.05). Further, HR collected during echocardiography revealed transient tachycardia 1 week after T10 contusion that was significantly greater than measurements made at the week ten time point (Table 2, Week 1 vs. Week 10, *P* = 0.042).

Timewise comparisons of T3 echocardiography revealed that contused rats had reduced left ventricular internal diameter during diastole (LVIDd) at five and 10 weeks postinjury compared to preinjury (Table 2, Preinjury vs. Week 5, *P* ≤ 0.001; Preinjury vs. Week 10, *P* ≤ 0.05). End‐diastolic volume (EDV) was also reduced at these time points (Fig. 6H; Preinjury vs. Week 5, *P* ≤ 0.001; Preinjury vs. Week 10, *P* ≤ 0.05), while end‐systolic volume (ESV) remained unchanged (Fig. 6I). Further, cardiac output (CO) was decreased at 5 weeks postinjury (Fig. 6F; Preinjury vs. Week 5, *P* ≤ 0.05), most likely due to a lower stroke volume (SV), although this only approached significance (Fig. 6D, E; SV, *P* = 0.06). Flow indices, left ventricular dimensions, and measures of diastolic function were similar to preinjury measurements at all time points assessed after T10 contusion.

### Influence of dobutamine on cardiac function in T3 contused animals

Prior to T3 contusion, Dobutamine infusion resulted in a dose‐dependent decrease in EDV and ESV, with a concurrent increase in ejection fraction (Table 2; EF). Flow indices (SV, HR, and CO) did not change significantly with increased Dobutamine concentrations (Table 2).

One and five weeks after T3 contusion, Dobutamine infusion resulted in a decrease in ESV with no change in EDV measurements (Table 2). Similar to preinjury assessments, T3 animals also experienced an increase in EF with increasing concentration of Dobutamine (Table 2). One week following T3 contusion, Dobutamine administration elicited a dose‐dependent increase in HR, SV, and CO (Table 2). The dose‐dependent increase in stroke volume was still present at 5 weeks postinjury. At 10 weeks following injury, Dobutamine induced dose‐dependent decreases in ESV and increases in EF much like preinjury measurements (Table 2). Unlike preinjury, however, T3 rodents responded to increasing concentrations of Dobutamine with increases in HR during terminal assessments (Table 2). Stroke volume and CO were unaffected at this later time point.

Hemodynamic responses to Dobutamine infusion are shown in Table 2. Systolic and mean blood pressures were not significantly affected by increasing concentrations of Dobutamine. Diastolic blood pressure, however was significantly lower at higher concentrations of Dobutamine during preinjury assessments and again at 10 weeks postcontusion.

### Influence of dobutamine on cardiac function in T10 contused animals

The effects of Dobutamine infusion on cardiac function following T10 contusion were similar to preinjury measurements. Average group results can be found in Table 2. Systolic, diastolic, and mean blood pressure were not significantly affected by increasing concentrations of Dobutamine infusion. Conversely, Dobutamine administration resulted in significantly higher HR measurements at the 30‐*μ*g dose prior to SCI and 1 week following injury.

Dobutamine also induced a dose‐dependent decrease in end‐systolic volume (ESV) with a concurrent increase in ejection fraction (EF) at all time points assessed. End‐diastolic volume (EDV) was also decreased with increasing concentrations of Dobutamine prior to SCI and at 5 weeks postcontusion. Like preinjury measurements, Dobutamine administration postcontusion resulted in a dose‐dependent increase in cardiac output (CO) (Table 2, Note, Week 5, *P* = 0.054 approaches significance). Stroke volume (SV) was also increased in the presence of Dobutamine at one and ten weeks after injury. There was a dose‐dependent reduction in the left ventricular internal diameter (LVID) during systole and diastole at all time points assessed.

## Discussion

### Summary of findings

These results demonstrate in a rodent model of incomplete SCI that both high (T3) and low thoracic (T10) contusion injuries result in abnormal CV control during the increased cardiopulmonary demand of an exercise challenge. Specifically, following moderately severe contusion, rats experience drops in HR acutely and large fluctuations in MBP sub‐acutely (weeks 2–5) and chronically (week 10) during an exercise challenge consisting of swimming multiple laps of a 1.5 m pool. This abnormal hemodynamic response to an exercise challenge, manifested as the inability to maintain BP control, progressively worsens with time postinjury. Importantly, deficits were not observed at rest, suggesting there is adequate residual sympathetic innervation to the CV system following moderately severe contusions to maintain MBP and HR in the absence of an exercise challenge.

Further, echocardiography revealed that cardiac structure and function was not significantly affected by either T3 or T10 thoracic contusions, even in the wake of reduced mobility and presumed partial unloading of the left ventricle. While there is attenuated systolic function in T3 animals at 5 weeks postinjury, this dysfunction is transient and largely recovers by 10 weeks. Thus, it is unlikely that cardiac decline is responsible for the poor BP control during an exercise challenge in our chronic contusion model. Instead, we speculate that the lack of hemodynamic stability during active exercise is due to abnormal autonomic control of vascular structures below the lesion.

### Maintenance of cardiovascular control during rest

Individuals with high thoracic SCI generally present with resting bradycardia and hypotension (West et al. 2012b, 2013). Bouts of OH and AD are also common, and can be a limiting factor in rehabilitation efforts (Harkema et al. 2008; Weaver et al. 2012). However, preclinical studies investigating the effects of high thoracic SCI on hemodynamic stability have provided varied results, most likely due to the heterogeneity of the study conditions. For instance, in agreement with clinical data, a study by West et al. (2015) showed that rats with complete transection of the upper thoracic spinal cord present with persistent hypotension beginning 1 week after injury. Conversely, we have shown that animals with either high or low thoracic contusion injuries can maintain BP within normal limits while at rest. This is in keeping with other reports that rodents with high incomplete SCI retain BP values that are not significantly different from preinjury measurements (Maiorov et al. 1998; Mayorov et al. 2001). This apparent disparity is likely due to spared sympathetic preganglionic neurons, and their inputs from medullary neurons, that are important for CV control. Thus, it appears that mimicking in the rat model the clinical observations made with human subjects at rest requires full transections or severe contusions that essentially eliminate descending input onto sympathetic preganglionic neurons in the upper thoracic spinal cord, despite the fact that most human injuries are anatomically incomplete (Kakulas 2004).

### Bradycardia during the exercise challenge: neurogenic shock?

Despite normal hemodynamic control at rest, animals in this study were unable to maintain CV control during an exercise challenge. Our findings suggest that the normally interdependent control of MBP and HR is decoupled acutely following injury, allowing changes in MBP to occur without compensatory changes in HR. In intact rodents the swimming exercise challenge induces an increase in HR, presumably associated with enhanced cardiac output in response to the increased metabolic demand of working musculature. At 1 week post‐T3 or T10 contusion, rats exhibited severe bradycardia during the exercise challenge along with increased MBP (although this was slightly blunted in T3 animals). Failure to increase HR in response to exercise acutely is likely due to neurogenic shock, a condition of hyporesponsiveness of the sympathetic nervous system (SNS). Although it has been reported previously that individuals with low thoracic injuries can experience abnormal CV control acutely (Guly et al. 2008; Mallek et al. 2012) neurogenic shock is generally thought to affect individuals with cervical and high thoracic lesions due to the disruption of medullary input onto sympathetic preganglionic neurons. However, our results suggest that bradycardia during an exercise challenge is independent of the lesion level and is due to an inability of the spinal cord circuitry to increase sympathetic output to the myocardium. In fact, MBP and HR recordings from T3 and T10 animals were surprisingly similar, implying that the lack of CV control in these two injury models has a common origin. This argument is supported by the actions of Dobutamine acutely following T3 contusion, serving as a surrogate for increased sympathetic tone to the heart that partially restored the chronotropic ability of the myocardium, essentially normalizing HR responses and flow indices.

### Progressive decline in hemodynamic control during exercise challenge

T3 and T10 injured animals displayed large oscillations in BP during the swimming exercise challenge (i.e., MBP Excursion) and these oscillations increased in amplitude over time. These rising phase of each oscillation occurred when the animal was swimming and the falling phase when the animal exited the water and was moved back to the far end of the pool. Few of these animals, regardless of injury level, had significant hindlimb movement during the swimming suggesting that that the oscillations were not the result of hindlimb muscle activation, but rather in response to forelimb movement without adequate compensation for rapid increases in cardiac output. The reason for this is likely multifactorial, but might involve changes in the peripheral vasculature due to partial denervation. Numerous groups have highlighted the rapid changes that occur in vascular structures following periods of immobility, including both human and rodent studies, which demonstrate diameter decreases in the femoral artery following periods of inactivity, such as SCI or lower limb immobilization (Sugawara et al. 2004; de Groot et al. 2006). Interestingly, increased pressure instability in our animals appeared to coincide with improved hindlimb performance during overground locomotion (BBB scale). This is counterintuitive given that exercise training and increased mobility have been shown to be beneficial to vascular function following SCI (Gerrits et al. 2001; Ditor et al. 2005; Thijssen et al. 2006). However, some of these studies included functional electrical stimulation (FES) of the lower extremities and clinical results are mixed with respect to the effects of such exercise on the ability of the vascular wall to accommodate changes in BP and flow. Given the increased activity of the hindlimbs during in‐cage locomotion after week 3, postinjury, reflected in BBB scores above 10 or 11, the structural characteristics of the vasculature may have returned to preinjury measures whereas sympathetic modulation of vascular tone remained attenuated.

The inability to maintain MBP consistently throughout the exercise challenge is a cause for concern given that large and/or rapid fluctuations in arterial pressure can damage the vascular endothelium, thereby increasing CVD risk (Fry 1968). The inability to control BP within a narrow range is a significant pathology that can negatively impact both cardiac and vascular structure and function. For instance, rodent studies examining the effects of recurrent bouts of AD show that unstable BP results in vascular hyper‐responsiveness to *α*‐adrenoceptor activation (Arnold et al. 1995; Alan et al. 2010). While the swimming exercise challenge in these studies does not elicit maximum pressure values that would be deemed clinically detrimental, the rapid swings in BP are nonetheless substantial (>30 mmHg or 25%) and deserves further investigation as repetitive rapid elevations in arterial pressure may induce shear injury to the vascular endothelium (Allen et al. 2014) potentially resulting in arterial remodeling and subsequent CV complications.

### Temporal assessment of cardiac structure and function in T3 rodents

Numerous studies, both clinical and preclinical, have shown that high thoracic and cervical SCI results in reduced systolic function (CO, SV, and EF) and bradycardia at rest (Kessler et al. 1986; West et al. 2014). The present results illustrate a progressive decline in resting cardiac function that reaches significance at 5 weeks postinjury but has recovered at 10 weeks post‐SCI. This finding is important, as most preclinical studies investigating cardiac structure and function post‐SCI conduct “terminal assessments” around the five or 6 week time point (West et al. 2014; DeVeau et al. 2018). If rodents, even those with incomplete injuries, experience improvements after that time point, it could have profound implications for individuals living with SCI, especially if the improvements correspond to changes in locomotor behavior or capacity.

In agreement with what others have noted in clinical settings, contusion injury to the upper thoracic spinal cord leads to decreased ventricular diameter and, subsequently, reduced EDV (Kessler et al. 1986; West et al. 2012a). Given the sustained reductions in EDV at later time points, it is reasonable to suggest that the reduction in ventricular diameter is due to chronic volume and pressure unloading of the heart (Levine et al. 1997; Perhonen et al. 2001). The reduction in sympathetic tone below the level of the lesion in conjunction with reduced hindlimb activity could lead to attenuated preload and SV, which would likely contribute to an altered Starling curve and impaired contractility over time (West et al. 2014). However, given that our animals have flow indices that are not different from preinjury measurements and the deposition of collagen is minimal, it appears as though left ventricular function is not different from preinjury at ten weeks postinjury. Therefore, the reduction in EDV could be a consequence of reduced sympathetic tone in vascular beds below the lesion, an increase in the heart/body mass ratio, and/or an attenuated venous return in the wake of reduced skeletal muscle pump activity and metabolic demands made by the lower limb musculature.

### Concluding remarks

Exercise and rehabilitation are important aspects of care in the SCI community. However, the timing and intensity of exercise and rehabilitation is critical and little is known about how an acutely injured system responds to cardiopulmonary challenges. Thus, the purpose of the present study was to determine if a newly injured autonomic nervous system was capable of eliciting appropriate CV responses to an exercise challenge and to investigate the differences between high (T3) and low (T10) spinal cord injuries that involve different components of the spinal autonomic circuitry. Here, we show that rodents with acute T3 and T10 contusion spinal cord injuries exhibit abnormal responses to exercise and are unable to effectively control MBP and HR during periods of increased cardiopulmonary demand. Further, we identified a distinct loss of control over MBP, observed as dynamic lap‐by‐lap swings of 20–25 mm of Hg that persist and even increase into the chronic phase and that are not different for the T3 and T10 animals. This persistent deficit in autonomic control is not evident at rest and likely involves peripheral vasculature because we found no differences in either cardiac structure or function for T3 and T10 injured animals, even during a Dobutamine challenge. Our results suggest that the application of acute training programs should be undertaken with care, and attention to the dynamic changes in blood pressure during exercise should be maintained even into the chronic post‐SCI phase.

## Conflict Of Interest

None declared.

## References

[phy213634-bib-0001] Alan, N. , L. M. Ramer , J. A. Inskip , S. Golbidi , M. S. Ramer , I. Laher , et al. 2010 Recurrent autonomic dysreflexia exacerbates vascular dysfunction after spinal cord injury. Spine J. 10:1108–1117. 21094471 10.1016/j.spinee.2010.09.018

[phy213634-bib-0002] Allen, R. P. , E. S. Schelegle , and S. H. Bennett . 2014 Diverse forms of pulmonary hypertension remodel the arterial tree to a high shear phenotype. Am. J. Physiol. Heart Circ. Physiol. 307:H405–H417. 24858853 10.1152/ajpheart.00144.2014 PMC4121648

[phy213634-bib-0003] Arnold, J. M. , Q. P. Feng , G. A. Delaney , and R. W. Teasell . 1995 Autonomic dysreflexia in tetraplegic patients: evidence for alpha‐adrenoceptor hyper‐responsiveness. Clin. Auton. Res. 5:267–270. 8563459 10.1007/BF01818891

[phy213634-bib-0004] Basso, D. M. , M. S. Beattie , and J. C. Bresnahan . 1995 A sensitive and reliable locomotor rating scale for open field testing in rats. J. Neurotrauma 12:1–21. 7783230 10.1089/neu.1995.12.1

[phy213634-bib-0005] Boot, C. R. , J. T. Groothuis , H. Van Langen , and M. T. Hopman . 2002 Shear stress levels in paralyzed legs of spinal cord‐injured individuals with and without nerve degeneration. J. Appl. Physiol. 92:2335–2340. 12015344 10.1152/japplphysiol.00340.2001

[phy213634-bib-0006] Brockway, B. P. , P. A. Mills , and S. H. Azar . 1991 A new method for continuous chronic measurement and recording of blood pressure, heart rate and activity in the rat via radio‐telemetry. Clin. Exp. Hypertens. A 13:885–895. 1773522 10.3109/10641969109042094

[phy213634-bib-0007] Cragg, J. J. , J. A. Stone , and A. V. Krassioukov . 2012 Management of cardiovascular disease risk factors in individuals with chronic spinal cord injury: an evidence‐based review. J. Neurotrauma 29:1999–2012. 22738320 10.1089/neu.2012.2313

[phy213634-bib-0008] DeVeau, K. M. , K. A. Harman , J. W. Squair , A. V. Krassioukov , D. Magnuson , and C. R. West . 2017 A comparison of passive hind‐limb cycling and active upper‐limb exercise provides new insights into systolic dysfunction following spinal cord injury. Am. J. Physiol. Heart Circ. Physiol. 313:H861–H870. ajpheart.00046.02017. 28710067 10.1152/ajpheart.00046.2017 PMC9925118

[phy213634-bib-0009] DeVeau, K. M. , E. K. Martin , N. T. King , A. Shum‐Siu , B. B. Keller , C. R. West , et al. 2018 Challenging cardiac function post‐spinal cord injury with dobutamine. Auton. Neurosci. 209:19–24. 28065654 10.1016/j.autneu.2016.12.005 PMC5481490

[phy213634-bib-0010] DeVivo, M. J. , and Y. Chen . 2011 Trends in new injuries, prevalent cases, and aging with spinal cord injury. Arch. Phys. Med. Rehabil. 92:332–338. 21353817 10.1016/j.apmr.2010.08.031

[phy213634-bib-0011] Ditor, D. S. , M. J. Macdonald , M. V. Kamath , J. Bugaresti , M. Adams , N. McCartney , et al. 2005 The effects of body‐weight supported treadmill training on cardiovascular regulation in individuals with motor‐complete SCI. Spinal Cord 43:664–673. 15968298 10.1038/sj.sc.3101785

[phy213634-bib-0012] Fry, D. L. 1968 Acute vascular endothelial changes associated with increased blood velocity gradients. Circ. Res. 22:165–197. 5639037 10.1161/01.res.22.2.165

[phy213634-bib-0013] Gerrits, H. L. , A. de Haan , A. J. Sargeant , H. van Langen , and M. T. Hopman . 2001 Peripheral vascular changes after electrically stimulated cycle training in people with spinal cord injury. Arch. Phys. Med. Rehabil. 82:832–839. 11387591 10.1053/apmr.2001.23305

[phy213634-bib-0014] Gonzenbach, R. R. , B. Zoerner , L. Schnell , O. Weinmann , A. K. Mir , and M. E. Schwab . 2012 Delayed anti‐nogo‐a antibody application after spinal cord injury shows progressive loss of responsiveness. J. Neurotrauma 29:567–578. 21815784 10.1089/neu.2011.1752

[phy213634-bib-0015] de Groot, P. C. , M. W. Bleeker , D. H. van Kuppevelt , L. H. van der Woude , and M. T. Hopman . 2006 Rapid and extensive arterial adaptations after spinal cord injury. Arch. Phys. Med. Rehabil. 87:688–696. 16635632 10.1016/j.apmr.2006.01.022

[phy213634-bib-0016] Guly, H. R. , O. Bouamra , and F. E. Lecky . 2008 The incidence of neurogenic shock in patients with isolated spinal cord injury in the emergency department. Resuscitation 76:57–62. 17688997 10.1016/j.resuscitation.2007.06.008

[phy213634-bib-0017] Harkema, S. J. , C. K. Ferreira , R. J. van den Brand , and A. V. Krassioukov . 2008 Improvements in orthostatic instability with stand locomotor training in individuals with spinal cord injury. J. Neurotrauma 25:1467–1475. 19118454 10.1089/neu.2008.0572 PMC2729458

[phy213634-bib-0018] Hou, S. , H. Duale , A. A. Cameron , S. M. Abshire , T. S. Lyttle , and A. G. Rabchevsky . 2008 Plasticity of lumbosacral propriospinal neurons is associated with the development of autonomic dysreflexia after thoracic spinal cord transection. J. Comp. Neurol. 509:382–399. 18512692 10.1002/cne.21771 PMC2536612

[phy213634-bib-0019] Hou, S. , H. Duale , and A. G. Rabchevsky . 2009 Intraspinal sprouting of unmyelinated pelvic afferents after complete spinal cord injury is correlated with autonomic dysreflexia induced by visceral pain. Neuroscience 159:369–379. 19146928 10.1016/j.neuroscience.2008.12.022 PMC3546483

[phy213634-bib-0020] Kakulas, B. A. 2004 Neuropathology: the foundation for new treatments in spinal cord injury. Spinal Cord 42:549–563. 15346131 10.1038/sj.sc.3101670

[phy213634-bib-0021] Kessler, K. M. , I. Pina , B. Green , B. Burnett , M. Laighold , M. Bilsker , et al. 1986 Cardiovascular findings in quadriplegic and paraplegic patients and in normal subjects. Am. J. Cardiol. 58:525–530. 3751915 10.1016/0002-9149(86)90027-5

[phy213634-bib-0022] Krassioukov, A. , and V. E. Claydon . 2006 The clinical problems in cardiovascular control following spinal cord injury: an overview. Prog. Brain Res. 152:223–229. 16198703 10.1016/S0079-6123(05)52014-4

[phy213634-bib-0023] Krenz, N. R. , and L. C. Weaver . 1998 Sprouting of primary afferent fibers after spinal cord transection in the rat. Neuroscience 85:443–458. 9622243 10.1016/s0306-4522(97)00622-2

[phy213634-bib-0024] Krishnan, A. , J. I. Pike , R. McCarter , A. L. Fulgium , E. Wilson , M. T. Donofrio , et al. 2016 Predictive models for normal fetal cardiac structures. J. Am. Soc. Echocardiogr. 29:1197–1206. 27773520 10.1016/j.echo.2016.08.019

[phy213634-bib-0025] Laird, A. S. , A. M. Finch , P. M. Waite , and P. Carrive . 2008 Peripheral changes above and below injury level lead to prolonged vascular responses following high spinal cord injury. Am. J. Physiol. Heart Circ. Physiol. 294:H785–H792. 18055525 10.1152/ajpheart.01002.2007

[phy213634-bib-0026] Lee, W. , T. Riggs , V. Amula , M. Tsimis , N. Cutler , R. Bronsteen , et al. 2010 Fetal echocardiography: z‐score reference ranges for a large patient population. Ultrasound Obstet. Gynecol. 35:28–34. 20014329 10.1002/uog.7483

[phy213634-bib-0027] Levine, B. D. , J. H. Zuckerman , and J. A. Pawelczyk . 1997 Cardiac atrophy after bed‐rest deconditioning: a nonneural mechanism for orthostatic intolerance. Circulation 96:517–525. 9244220 10.1161/01.cir.96.2.517

[phy213634-bib-0028] Lujan, H. L. , H. Janbaih , and S. E. DiCarlo . 2012 Dynamic interaction between the heart and its sympathetic innervation following T5 spinal cord transection. J. Appl. Physiol. 113:1332–1341. 22723636 10.1152/japplphysiol.00522.2012 PMC3472491

[phy213634-bib-0029] Magnuson, D. S. , R. Lovett , C. Coffee , R. Gray , Y. Han , Y. P. Zhang , et al. 2005 Functional consequences of lumbar spinal cord contusion injuries in the adult rat. J. Neurotrauma 22:529–543. 15892599 10.1089/neu.2005.22.529

[phy213634-bib-0030] Maiorov, D. N. , M. G. Fehlings , and A. V. Krassioukov . 1998 Relationship between severity of spinal cord injury and abnormalities in neurogenic cardiovascular control in conscious rats. J. Neurotrauma 15:365–374. 9605350 10.1089/neu.1998.15.365

[phy213634-bib-0031] Mallek, J. T. , K. Inaba , B. C. Branco , C. Ives , L. Lam , P. Talving , et al. 2012 The incidence of neurogenic shock after spinal cord injury in patients admitted to a high‐volume level I trauma center. Am. Surg. 78:623–626. 22546142

[phy213634-bib-0032] Mayorov, D. N. , M. A. Adams , and A. V. Krassioukov . 2001 Telemetric blood pressure monitoring in conscious rats before and after compression injury of spinal cord. J. Neurotrauma 18:727–736. 11497098 10.1089/089771501750357663

[phy213634-bib-0033] Nash, M. S . 2005 Exercise as a health‐promoting activity following spinal cord injury. J. Neurol. Phys. Ther. 29:87–103, 106. 16386165 10.1097/01.npt.0000282514.94093.c6

[phy213634-bib-0034] Perhonen, M. A. , F. Franco , L. D. Lane , J. C. Buckey , C. G. Blomqvist , J. E. Zerwekh , et al. 2001 Cardiac atrophy after bed rest and spaceflight. J. Appl. Physiol. 91:645–653. 11457776 10.1152/jappl.2001.91.2.645

[phy213634-bib-0035] Plante, E. , D. Lachance , M. C. Drolet , E. Roussel , J. Couet , and M. Arsenault . 2005 Dobutamine stress echocardiography in healthy adult male rats. Cardiovasc. Ultrasound 3:34. 16250913 10.1186/1476-7120-3-34 PMC1276802

[phy213634-bib-0036] Radovits, T. , A. Olah , A. Lux , B. T. Nemeth , L. Hidi , E. Birtalan , et al. 2013 Rat model of exercise‐induced cardiac hypertrophy: hemodynamic characterization using left ventricular pressure‐volume analysis. Am. J. Physiol. Heart Circ. Physiol. 305:H124–H134. 23645462 10.1152/ajpheart.00108.2013

[phy213634-bib-0037] Smith, R. R. , D. A. Burke , A. D. Baldini , A. Shum‐Siu , R. Baltzley , M. Bunger , et al. 2006a The Louisville swim scale: a novel assessment of hindlimb function following spinal cord injury in adult rats. J. Neurotrauma 23:1654–1670. 17115911 10.1089/neu.2006.23.1654 PMC2833969

[phy213634-bib-0038] Smith, R. R. , A. Shum‐Siu , R. Baltzley , M. Bunger , A. Baldini , D. A. Burke , et al. 2006b Effects of swimming on functional recovery after incomplete spinal cord injury in rats. J. Neurotrauma 23:908–919. 16774475 10.1089/neu.2006.23.908 PMC2831776

[phy213634-bib-0039] Squair, J. W. , K. M. DeVeau , K. A. Harman , M. S. Poormasjedi‐Meibod , B. Hayes , J. Liu , et al. 2018 Spinal cord injury causes systolic dysfunction and cardiomyocyte atrophy. J. Neurotrauma 35:424–434. 28599602 10.1089/neu.2017.4984 PMC9836687

[phy213634-bib-0040] Sugawara, J. , K. Hayashi , F. Kaneko , H. Yamada , T. Kizuka , and H. Tanaka . 2004 Reductions in basal limb blood flow and lumen diameter after short‐term leg casting. Med. Sci. Sports Exerc. 36:1689–1694. 15595288 10.1249/01.mss.0000142410.45142.28

[phy213634-bib-0041] Thijssen, D. H. , P. Heesterbeek , D. J. van Kuppevelt , J. Duysens , and M. T. Hopman . 2005 Local vascular adaptations after hybrid training in spinal cord‐injured subjects. Med. Sci. Sports Exerc. 37:1112–1118. 16015126 10.1249/01.mss.0000170126.30868.fb

[phy213634-bib-0042] Thijssen, D. H. , R. Ellenkamp , P. Smits , and M. T. Hopman . 2006 Rapid vascular adaptations to training and detraining in persons with spinal cord injury. Arch. Phys. Med. Rehabil. 87:474–481. 16571385 10.1016/j.apmr.2005.11.005

[phy213634-bib-0043] Thijssen, D. H. , P. C. De Groot , A. van den Bogerd , M. Veltmeijer , N. T. Cable , D. J. Green , et al. 2012 Time course of arterial remodelling in diameter and wall thickness above and below the lesion after a spinal cord injury. Eur. J. Appl. Physiol. 112:4103–4109. 22526250 10.1007/s00421-012-2400-2 PMC3496545

[phy213634-bib-0044] Van Loan, M. D. , S. McCluer , J. M. Loftin , and R. A. Boileau . 1987 Comparison of physiological responses to maximal arm exercise among able‐bodied, paraplegics and quadriplegics. Paraplegia 25:397–405. 3684324 10.1038/sc.1987.70

[phy213634-bib-0045] Weaver, L. C. , J. C. Fleming , C. J. Mathias , and A. V. Krassioukov . 2012 Disordered cardiovascular control after spinal cord injury. Handb. Clin. Neurol. 109:213–233. 23098715 10.1016/B978-0-444-52137-8.00013-9

[phy213634-bib-0046] West, C. R. , I. G. Campbell , R. E. Shave , and L. M. Romer . 2012a Resting cardiopulmonary function in Paralympic athletes with cervical spinal cord injury. Med. Sci. Sports Exerc. 44:323–329. 21720277 10.1249/MSS.0b013e31822b7441

[phy213634-bib-0047] West, C. R. , P. Mills , and A. V. Krassioukov . 2012b Influence of the neurological level of spinal cord injury on cardiovascular outcomes in humans: a meta‐analysis. Spinal Cord 50:484–492. 22391687 10.1038/sc.2012.17

[phy213634-bib-0048] West, C. R. , A. Bellantoni , and A. V. Krassioukov . 2013 Cardiovascular function in individuals with incomplete spinal cord injury: a systematic review. Top. Spinal. Cord Inj. Rehabil. 19:267–278. 24244092 10.1310/sci1904-267 PMC3816721

[phy213634-bib-0049] West, C. R. , M. A. Crawford , M. S. Poormasjedi‐Meibod , K. D. Currie , A. Fallavollita , V. Yuen , et al. 2014 Passive hind‐limb cycling improves cardiac function and reduces cardiovascular disease risk in experimental spinal cord injury. J. Physiol. 592:1771–1783. 24535438 10.1113/jphysiol.2013.268367 PMC4001751

[phy213634-bib-0050] West, C. R. , D. Popok , M. Crawford , and A. V. Krassioukov . 2015 Characterizing the temporal development of cardiovascular dysfunction in response to spinal cord injury. J. Neurotrauma 32:922–930. 25630034 10.1089/neu.2014.3722

[phy213634-bib-0051] West, C. R. , M. A. Crawford , I. Laher , M. S. Ramer , and A. V. Krassioukov . 2016 Passive hind‐limb cycling reduces the severity of autonomic dysreflexia after experimental spinal cord injury. Neurorehabil. Neural Repair 30:317–327. 26159931 10.1177/1545968315593807

[phy213634-bib-0052] Whiteneck, G. G. , S. W. Charlifue , H. L. Frankel , M. H. Fraser , B. P. Gardner , K. A. Gerhart , et al. 1992 Mortality, morbidity, and psychosocial outcomes of persons spinal cord injured more than 20 years ago. Paraplegia 30:617–630. 1408338 10.1038/sc.1992.124

